# New frontiers in proton therapy: applications in cancers

**DOI:** 10.1186/s40880-019-0407-3

**Published:** 2019-10-22

**Authors:** Tai-Ze Yuan, Ze-Jiang Zhan, Chao-Nan Qian

**Affiliations:** 1 Department of Radiation Oncology, Guangzhou Concord Cancer Center, Guangzhou, 510045 Guangdong P. R. China; 2 Department of Radiation Oncology, Cancer Center of Guangzhou Medical University, Guangzhou, 510095 Guangdong P. R. China

**Keywords:** Proton therapy, Radiation therapy, Intensity-modulated, Systematic review, Cancer

## Abstract

Proton therapy offers dominant advantages over photon therapy due to the unique depth-dose characteristics of proton, which can cause a dramatic reduction in normal tissue doses both distal and proximal to the tumor target volume. In turn, this feature may allow dose escalation to the tumor target volume while sparing the tumor-neighboring susceptible organs at risk, which has the potential to reduce treatment toxicity and improve local control rate, quality of life and survival. Some dosimetric studies in various cancers have demonstrated the advantages over photon therapy in dose distributions. Further, it has been observed that proton therapy confers to substantial clinical advantage over photon therapy in head and neck, breast, hepatocellular, and non-small cell lung cancers. As such, proton therapy is regarded as the standard modality of radiotherapy in many pediatric cancers from the technical point of view. However, due to the limited clinical evidence, there have been concerns about the high cost of proton therapy from an economic point of view. Considering the treatment expenses for late radiation-induced toxicities, cost-effective analysis in many studies have shown that proton therapy is the most cost-effective option for brain, head and neck and selected breast cancers. Additional studies are warranted to better unveil the cost-effective values of proton therapy and to develop newer ways for better protection of normal tissues. This review aims at reviewing the recent studies on proton therapy to explore its benefits and cost-effectiveness in cancers. We strongly believe that proton therapy will be a common radiotherapy modality for most types of solid cancers in the future.

## Introduction

Research on the role of radiotherapy in the management of cancer has been intensified in the last 2 decades [1–3]. During the whole course of disease treatment, 60%–70% of all cancer patients need to undergo radiotherapy [1], mostly with photon therapy, which is delivered with linear accelerators. As the latest research and application of radiotherapeutics, heavy ion therapy especially proton therapy is well-known for its multitude of advantages over photon therapy due to its physical characteristics, known as Bragg peak. Phase II clinical trials on boron neutron capture therapy (BNCT), a binary therapeutic modality based on the nuclear capture and fission reactions that occur when the stable isotope boron-10 is irradiated with neutrons to produce high-energy alpha particles and recoiling lithium-7 nuclei, are being carried out [4, 5]. With low entrance dose and no exiting dose, protons deposit most energy in a certain depth which is near the end of the penetration path, known as the Bragg peak. Because of the favorable feature, lots of patients diagnosed with cancer have been treated with proton therapy for the last 30 years worldwide [6].

Initially, proton therapy was used to treat radio-resistant tumors such as chordoma and melanoma. With the development of delivery technique, indications were gradually expanded to other cancers, such as pediatric, head and neck, lung, liver, pancreatic, and prostate cancers. Although accompanied with high investment and running costs, proton therapy centers have increased quickly since the first hospital-based Loma Linda University Proton Therapy Center (Loma Linda city, California, USA) was established in 1990. Now there are ~ 70 proton therapy centers worldwide and more than 190,000 patients have been treated with proton therapy. The existence of these centers enables large cooperative clinical trials to be performed; significantly increasing the scientific literature on proton therapy during the last decade.

In the present article, we review the recent studies on proton therapy in order to explore its benefits, value and cost-effectiveness in various cancers.

## Advantages of proton physical characteristics

As a kind of charged particles, protons can penetrate a certain depth in tissues which depends on the energy of proton. Proton has physical advantages over photon by depositing the majority of its energy at the site of “Bragg Peak”, beyond which there is no energy delivered [7]. Hence, normal tissues distal to the Bragg peak can be protected by avoiding radiation doses. At the same time, comparing to the most advanced photon techniques such as intensity-modulated radiotherapy (IMRT) [8] and volumetric modulated arc therapy (VMAT), proton therapy can deliver similar or higher radiation doses to tumor target volumes with a 50%–60% reduction in integral or “total body” radiation dose [9]. With the development of pencil beam scanning technique, the newest generation of proton equipment can also perform intensity-modulated proton therapy (IMPT) which yields highly conformal dose distribution around the target volumes [10]. Because of these characteristics, proton therapy has become the optimal radiotherapy for pediatric cancer patients and is being actively studied for various tumor types in adults.

## Role of proton therapy in cancer treatments

Currently, indications of proton therapy include pediatric, head and neck, lung, liver, pancreatic, and prostate cancers [11]. Proton therapy can increase radiotherapeutic ratio. The criteria to choose proton therapy depends on whether it delivers a higher dose to targeted volumes while avoiding maximum dose constraints to organs at risk or similar dose while significantly decreasing the irradiated doses to organs at risk. Thus, cancers that are close to serial organs at risk are likely to benefit from proton therapy. Those include chordoma, nasopharyngeal carcinoma, para-nasal sinus cancer, and intracranial tumors. On the other hand, cancers that are near parallel organs at risk could also benefit from proton therapy because parallel organs at risk are sensitive to the irradiated volumes, which can be reduced dramatically by proton therapy.

Many dosimetric studies and clinical data have shown the advantages of proton therapy over photon therapy. Generally, proton therapy is associated with higher tumor dose distribution and/or lower toxicities, which is discussed, organ by organ, as following.

### Head and neck cancers

Radiotherapy is an important treatment modality for head and neck cancer. Compared to photon therapy which includes the latest technologies such as VMAT and IMRT, proton therapy has shown an advantage for protecting the brain stem, salivary glands, spinal cord, and larynx [8, 9, 12–14].

Several recent dosimetric studies have confirmed the dose reduction to normal tissues using proton therapy for oropharyngeal carcinoma, compared with IMRT [15–17]. In one study, Holliday et al. [15] reported that there were significantly lower doses to the brain stem, cerebellum, posterior oral cavity, pharyngeal constrictors and the esophagus in proton therapy plans compared with IMRT plans using a case-matched control analysis. However, not every head and neck cancer patient could benefit from proton therapy due to tumor size and the relationship between the tumor and the surrounding organs at risk. So, comparative dosimetric planning needs to be done for each patient to choose the best technique to be applied. Jakobi et al. [12] evaluated the dose distribution of IMRT compared to IMPT in 45 patients with locally advanced head and neck cancer based on a Normal Tissue Complication Probability (NTCP) model. The analysis of differences in NTCP reduction by IMPT demonstrated a higher benefit of proton therapy in reducing dysphagia for patients with tumors in the upper head and neck area.

Further, the clinical benefit of proton therapy for head and neck cancers were recently reviewed by Blanchard et al. [18]. To compare the clinical efficacy and potential for toxicity reduction of proton therapy with photon therapy, a propensity-matched retrospective study on 164,580 patients with head and neck cancer was performed. It showed that proton therapy (*n* = 157) was associated with an improved 5-year overall survival (OS) compared with photon therapy (66.8% vs. 60.0%, *n* = 1400, Hazard ratio [HR], 0.73, *P* = 0.028), respectively [19]. However, proton therapy had a similar 5-year OS to IMRT treatment (*n* = 469), which was 66.8% and 64.0% (HR, 0.78, *P* = 0.14), respectively. More prospective randomized studies are necessary to deepen our understandings on the potential benefits of proton therapy.

As a common head and neck cancer in Southern China, nasopharyngeal carcinoma (NPC) is a good indication of proton therapy because of its special anatomy location and close proximity to the eyes and cranial nerves [20, 21]. The advantage on dosimetry for proton therapy over IMRT is a dramatic volume reduction of normal tissue receiving low- to medium-radiation doses [22–24]. Lewis et al. [23] reported his findings on 9 NPC patients who were treated with proton therapy and concurrent cisplatin-based chemotherapy. After follow-up (median: 24.5 months), their 2-year local control (LC) and OS was 100% and 88.9%, respectively. The most common acute grade 3 toxicity was dermatitis, observed in 4 patients. No patients had acute grade 4 or 5 toxicities. All 9 patients developed mucositis, of grade 2 in 8 patients and grade 3 in 1 patient. These shows demonstrate good clinical evidences of the encouraging outcomes for using proton therapy with low adverse event, similar to prior reports of IMRT [25]. Further randomized studies are needed to fully elucidate the extent of the observed advantages of proton therapy on dosimetry translating to reduced toxicity and improve survival.

### Breast cancer

A meta-analysis showed that adjuvant breast radiation therapy after breast-conserving surgery reduces local and metastatic relapses and decreases the cancer-specific death rate with an absolute benefit of 3.8%, from 25.2% to 21.4%, after a 15-year follow-up [26]. However, long term cardiovascular toxicities and second cancers induced by radiation therapy will counteract the benefit on OS [27, 28].

Compared to IMRT, proton therapy beam scanning was found to potentially reduce the mean heart dose close to 0–0.5 Gy for left-sided breast cancer [29], which makes it possible to cover the internal mammary node in the target for breast cancer radiotherapy without a significant dose to the heart. Other studies [30, 31] also confirmed that proton therapy possessed a better dose distribution profile and reduced mean heart dose compared with IMRT. Another advantage of proton therapy is that it can dramatically decrease the volume of normal tissue receiving low radiation dose, which could also lead to a lower incidence of secondary malignancy. Several studies [29, 32, 33] on proton therapy for breast cancer have demonstrated a reduction of irradiated volumes in normal tissue.

Cuaron et al. [34] respectively analyzed the early toxicity data for breast cancer patients treated with postoperative proton therapy. Among patients with > 3 months of follow-up (*n* = 28), grade 2 dermatitis occurred in 20 patients (71.4%), with 8 (28.6%) experiencing moist desquamation and 1 (3.6%) with grade 3 reconstructive complications. To further determine the toxicity of proton therapy, an ongoing prospective phase II trial (NCT01758445) on patients with stage II/III breast cancers has been undertaken, aiming to mainly evaluate its related cardio-vascular adverse effects.

### Non-small cell lung cancer (NSCLC)

The dose–effect relationship is well-demonstrated in various cancers. A higher dose is related to a higher local control of the tumor and better disease-free survival (DFS) [35]. However, a recent clinical trial (RTOG 0617 trial) [36] on NSCLC with photon therapy did not observe better survival after using a higher dose (74 Gy). The main reason was that radiation-induced heart disease leads to more death in the high-dose arm since the mean dose to the heart in this trial was an independent prognostic factor for OS.

Compared to photon therapy such as IMRT/VMAT, proton therapy for NSCLC could deliver a higher dose to target volumes while decreasing dose to organs at risk, which makes it possible to attain better local control and survival [37, 38].

Criticism of proton therapy for lung cancer comes from the uncertainties related to the respiratory movement and tissue density which could dramatically affect the range of proton. However, Chang et al. [39] confirmed the feasibility of proton therapy for lung cancer using a 4-dimensional computed tomography (4DCT) to delineate an internal gross tumor volume and expanding a margin of 5 mm to form planning target volume. No grade 4 or 5 toxicities were observed in this study after a median follow-up of 6.5 months.

Nguyen et al. [40] reported the long-term results of proton therapy in 134 NSCLC patients with a 4.7-year median follow-up. The median OS was 30.4 months in stage III patients with 1 (0.7%) grade 4 and 16 (11.9%) grade 3 toxicities, and similar promising results were also reported in other studies [41–44]; in one of which, Chang et al. [44] recently published the long-term results of their prospective phase II study with a median follow up of 27.3 months for all patients and 79.6 months for the survivors. The median OS was 26.5 months with a 5-year progression-free survival (PFS) of 22%. Local recurrences occurred in 16% of patients, whereas distant metastases occurred in 48%. There was no acute grade 4 and 5 pneumonitis reported. However, acute grade 3 esophagitis occurred in 8% of patients. Furthermore, late toxicities included grade 3 pneumonitis (16%), grade 4 bronchial fistula (2%) and grade 4 esophagitis (2%).

Liao et al. [45] reported that there was no clinical difference between IMRT and proton therapy for NSCLC (passive scattering technique). Thus, further clinical trial and optimization of proton therapy technique, particularly IMPT, is still needed.

### Hepatocarcinoma

It has been a great challenge to deliver radical dose to hepatocellular carcinoma due to radiation-sensitivity of liver tissue and loco-regional invasion of the tumor even with IMRT. An important margin has also to be added (approximately 1–2 cm) taking into account the mobility of abdominal organs in traditional photon radiotherapy. A dosimetric study has shown that IMPT could decrease the dose delivered to organs at risk compared with VMAT [46], allowing the possibility to increase the dose given to the tumor without increasing radiation-induced hepatic toxicities.

In operable hepatocarcinoma patients, due to their underlying poor performance status and associated comorbidities, they can benefit from local treatments such as stereotactic radiotherapy which can yield up to 90% of local control [47]. However, for large tumors > 5 cm or specific anatomic situations (i.e. hepatic hilum, central tumor) are not eligible for these local photon therapies. In these settings, proton therapy has proved its ability to deliver higher doses to target volumes without increasing the risk of hepatic toxicities [48, 49]. A retrospective study [50] on 22 patients with large hepatocellular carcinoma (median size: 11 cm, range: 10–14 cm) treated with proton therapy (72.6 Gy) demonstrated promising result with a 2-year LC of 87%, 2-year OS of 36%, 2-year PFS 24% and no grade 3–5 late toxicities. Furthermore, a multi-institutional phaseIIclinical trial [51] investigated the efficacy and safety of proton therapy for hepatocarcinoma. With a median follow-up of 19.5 months, the 2-year LC and OS were 94.8% and 63.2%, respectively. Four patients (4.8%) experienced at least 1 grade-3 radiation-related toxicity, such as fatigue, rash, and nausea. There was no grade-4 or grade-5 radiation-related toxicity.

Several studies evaluating the role of proton therapy, transarterial chemoembolization (TACE), and radiofrequency ablation for hepatocarcinoma are ongoing. Recently, a randomized trial [52] on TACE versus proton therapy for hepatocarcinoma showed a trend toward improved 2-year PFS (31% vs. 48%, *P* = 0.06) and 2-year LC (45% vs. 88%, *P* = 0.06) favoring proton therapy, although the difference was not statistically significant. Additionally, there are many ongoing single-arm clinical trials on proton therapy for hepatocarcinoma in specific clinical setting such as inoperable disease and portal vein tumor thrombus. The results of these trials will improve the level of evidence for the clinical efficacy of treatment using proton therapy in hepatocarcinoma.

### Prostate cancer

The role of proton therapy for prostate cancer has been controversial. Several dosimetric studies have demonstrated that proton therapy for prostate cancer could lower the mean dose to the rectum and bladder compared to VMAT [53–55]. However, in terms of high dose volume, proton did not have obvious advantages over photon therapy due to the anatomic location of the rectum and bladder. It was noted that proton therapy only treated primary prostate without irradiating regional lymph nodes.

Clinically, Takagi et al. [56] reported the long-term outcomes of prostate cancer patients treated with proton therapy. In total, 99% of the patients received a dose of 74 Gy with a median follow-up of 70 months. For the low-, intermediate-, high-, and very high-risk groups, the 5-year failure-free biological recurrence was 99%, 91%, 86%, and 66%, respectively, and the 5-year cancer-specific survival was 100%, 100%, 99%, and 95%, respectively. Furthermore, grade 2 or higher late gastrointestinal and genitourinary toxicities were 3.9% and 2.0%, which was supported by other studies which also found a low rate of gastrointestinal toxicity [57]. However, until now, there is no high-level evidence-based study to suggest that proton therapy is superior to photon therapy in regards to prostate cancer control and toxicities.

### Pediatric cancer

Due to the improved survival of pediatric cancer patients over the past 10 years, more attention has been paid on decreasing long-term side effects to improve patients’ quality of life. It was reported that > 60% of these cancer survivors will experience one or more radiation-related late toxicities and many of these adverse events would be life-threatening [58].

It is well demonstrated that proton therapy can spare many normal tissues and reduce the integral dose to organs at risk. A meta-analysis [58] with 650 patients in 23 primary studies showed that proton therapy could reduce the radiation dose to normal tissues.

It was found that the outcomes of survival and tumor control in proton therapy for treating pediatric patients diagnosed with central nervous system cancer were comparable to that of photon therapy [59]. The incidence rate of severe acute and late toxicities was reduced with the use of proton therapy. Furthermore, the severity of endocrine, neurological, intelligence quotient and quality of life deficits was also decreased. Extensive follow-up is necessary to validate the incidences of late toxicities and secondary malignancies. To date, evidence on proton therapy for pediatric cancer patients supports its clinical effectiveness and potential benefits in reducing late toxicities in later life. Besides, high-quality clinical research in proton therapy is still highly needed [58].

### Re-irradiation

Tumor recurrence is one of the main treatment-failure after radiotherapy and is usually unresectable because of different factors. In these cases, re-irradiation with photons is an important therapeutic option. However, due to the organs at risk constraints, a full-dose re-irradiation is rarely achievable. Thus, the local control rate is poorer compared to a primary tumor irradiation. Proton therapy is a highly accurate radiotherapy technique, which is a good option for delivering a high dose to target volumes to improve local control while sparing the surrounding critical normal tissue. Published studies on re-irradiation with proton therapy have shown promising results. Phan et al. [60] reported their findings on treating 60 recurrent head and neck cancer patients with proton therapy (median dose: 66 Gy). After a median follow-up time of 13.6 months, their observed 1-year locoregional recurrence free survival (LRFS), OS, PFS, and DFS were 68.4%, 83.8%, 60.1%, and 74.9%, respectively. Eighteen patients developed acute grade 3 toxicity and 3 patients may have died of reirradiation-related toxicities.

A study on the reirradiation of thoracic cancers with IMPT [61] demonstrated that IMPT could provide durable local control with minimal toxicity, with a 1-year LRFS and PFS of 84% and 76%, respectively. Despite that 2 patients developed late grade 3 pulmonary toxicity, none had grade 4–5 toxicities.

## Cost-effectiveness analysis

More than 50% of cancer patients were reported to undergo radiotherapy during their whole disease treatment [62]. Due to the dosimetric benefits of proton, the clinical use of proton therapy for cancers is dramatically growing and more than 79 operational facilities worldwide by 2019 [63]. Currently, proton therapy is being used for the radiation therapy of pediatric cancer, head and neck, lung, hepatocellular, breast and prostate cancer. However, considering cost-effectiveness, the significance of proton therapy has been controversial.

A Swedish study [64] evaluated the cost-effectiveness of proton therapy and photon therapy for medulloblastoma by Markov modeling. Compared to photon therapy, the initial cost of proton therapy was 2.4-fold higher ($12,364 vs. $5129). However, the costs of adverse effects in proton therapy and photon therapy were $5121 and $40,967, respectively, rendering the total costs of proton therapy and photon therapy being $17,484 and $46,096, respectively (2.6-fold decrease for proton therapy). Similar observations were also observed in other studies [65, 66]. Therefore, proton therapy was thought to be the most cost-effective option for brain tumors [67]. For head and neck cancer patients, recent data showed up to 50% reduction in the use of gastrostomy feeding tubes with proton therapy compared to that with IMRT [68] and Markov modeling also showed proton therapy offered superior cost-effectiveness.

For locally advanced NSCLC, Lievens et al. [69] demonstrated that proton therapy increased the quality-adjusted life-years (QALYs) gained by 0.549 and 0.452 compared with 3-dimensional conformal radiotherapy (3DCRT) and IMRT, which means that those patients had cost-effectiveness benefits. However, in inoperable stage I NSCLC, stereotactic body radiotherapy (SBRT) was identified as the cheapest at $4501 compared with 3DCRT and proton therapy for $9862 and $19,469, respectively. So, cost-effectiveness benefits are low in this setting [67].

In left-sided breast cancer, there is a good indication for the use of proton therapy because proton therapy could deliver lower dose to heart and decrease the risk of cardiovascular disease. For those patients, initial radiotherapy costs were $13,610 for proton therapy and $6051 for whole breast photon therapy [70]. Considering that cardiac disease average cost ($80,596 in whole breast photon therapy vs. $41,491 in proton therapy), proton therapy were favorable for appropriately selected patients with left-sided cancers at high-risk of cardiac toxicity compared with whole-breast photon therapy.

## Conclusion

Proton therapy has dosimetric advantages over photon therapy of superior normal tissue sparing, particularly in the low to moderate dose range, which makes it possible to achieve higher tumoricidal dose. Furthermore, IMPT offers a crucial capability to balance normal tissue and tumor doses. In the past decades, despite the fact that 190,000 patients have been treated with proton therapy, which offers superior cost-effectiveness in medulloblastoma, head and neck cancer and left-sided breast cancer, further research is still necessary to demonstrate the potential of proton therapy in other cancers.

## Data Availability

References [6, 63] were searched from the official website of Particle Therapy Co-Operative Group. The other data presented in this manuscript have been published and can be retrieved by going to the references indicated. There are no materials relating to this review.

## Abbreviations

BNCT: boron neutron capture therapy
IMRT: intensity-modulated radiotherapy
VMAT: volumetric modulated arc therapy
IMPT: intensity-modulated proton therapy
NTCP: Normal Tissue Complication Probability
OS: overall survival
HR: hazard ratio
NPC: nasopharyngeal carcinoma
LC: local control
NSCLC: non-small cell lung cancer
DFS: disease-free survival
4DCT: 4-dimensional computed tomography
PFS: progression-free survival
TACE: transarterial chemoembolization
LRFS: locoregional recurrence free survival
QALY: quality-adjusted life-year
3DCRT: 3-dimensional conformal radiotherapy
SBRT: stereotactic body radiotherapy
